# The impact of Propolis on catheter exit site infection and peritonitis in peritoneal Dialysis patients: a clinical trial

**DOI:** 10.1186/s12882-022-03036-7

**Published:** 2022-12-23

**Authors:** Lila Moghiseh, Monir Nobahar, Raheb Ghorbani, Shiva Sirafian

**Affiliations:** 1 Nursing Care Research Center, Semnan University of Medical Sciences, Semnan, Iran; 2 Social Determinants of Health Research Center, Semnan University of Medical Sciences, Semnan, Iran; 3 Department of Nursing, Faculty of Nursing and Midwifery, Semnan University of Medical Sciences, Semnan, Iran; 4 Nursing Care Research Center and Social Determinants of Health Research Center, Semnan University of Medical Sciences, Semnan, 3513138111 Iran; 5 Department of Epidemiology and Biostatistics, Faculty of Medicine, Semnan University of Medical Sciences, Semnan, Iran; 6 Isfahan Kidney Diseases Research Center, Internal Medicine Department, Isfahan University of Medical Sciences, Isfahan, Iran

**Keywords:** Catheter exit site infection, Clinical trial, Peritoneal Dialysis patients, Peritonitis, Propolis

## Abstract

**Background:**

Peritonitis is one of the major complications of peritoneal dialysis. The most common cause of peritonitis is infection at the catheter exit site. This study aimed to determine the effect of propolis on the incidence of catheter exit site infection and peritonitis in peritoneal dialysis patients.

**Method:**

This study was a double-blind clinical trial (2019–2020) with peritoneal dialysis patients. Ninety peritoneal dialysis patients were allocated to three groups (placebo, control, intervention) using block randomization method. Catheter exit site was washed with 0.9% normal saline and dressing was done every other day after the morning peritoneal dialysis exchange by use of normal saline in placebo, mupirocin in control, and propolis in intervention group, for 6 months.

**Discussion:**

10% of the patients in the placebo and 6.7% in the control group developed catheter Exit Site Infection, but none patient in the intervention group developed this infection (*P* = 0.469). Whereas 6.7% in both the placebo and control groups developed peritonitis, but none patient in the intervention group contracted peritonitis (*P* = 0.997). No significant differences in the incidence of catheter exit site infection and peritonitis among the three groups were observed. Considering that mupirocin is of chemical origin and may lead to drug resistance whereas propolis is of plant origin and does not produce drug resistance, the use of propolis is recommended.

**Trial registration:**

Iranian Registry of Clinical Trials [IRCT20110427006318N10] (17/01/2019).

## Background

The loss of renal function due to decreased nephron function is called Chronic Kidney Disease (CKD) [1]. With the rising prevalence of diabetes mellitus, hypertension, obesity, and metabolic syndrome in recent decades, there has been a sharp increase in the prevalence of CKD [2]. It has been predicted that by the end of 2020, the number of CKD patients will reach 4 million. According to the Iran Consortium of Dialysis (ICD) report the average prevalence of CKD in Iran is 680 per million, which is higher than the global average (510 per million), 58,000 patients treated with one of renal replacement therapy (RRT), Hemodialysis was the primary treatment of the patient with almost 50%, transplantation 47% and peritoneal dialysis (PD) 3% [3].

PD is highly regarded because of its simplicity, easy access, and reasonable cost [4]. The most important complication of PD is peritonitis, which may leads to the discontinuation of this treatment [5]. In most cases, one of the main causes of peritonitis is the catheter Exit Site Infection (ESI) [6]. Peritonitis can be diagnosed based on two groups of symptoms: clinical and laboratory. Clinical symptoms of peritonitis include abdominal pain and PD cloudy effluent and its laboratory signs are dialysate effluent leukocyte count exceeding 100 with at least 50% polymorphonuclear leukocytes and positive culture of PD effluent [7]. Peritonitis can lead to hospitalization, increased treatment costs, long-term peritoneal side effects, and death [8].

According to the recommendations of the International Society for Peritoneal Dialysis (ISPD), the key measure for the prevention and treatment of peritonitis include the use of prophylactic antibiotics before placing the PD catheter, daily use of topical antibiotic cream or ointment at the catheter exit site, and rapid treatment of exit-site infections. Upon the detection of PD-related peritonitis, after taking microbiological samples, antibiotics for gram-positive and gram-negative organisms (including Pseudomonas species) should be initially administered preferably intraperitoneally [9].

Considering the side effects, inconsistent efficacy, and high cost of chemical treatments, recent years have seen a growing interest in using complementary therapies and traditional methods whenever possible [10]. Complementary therapies to describe interventions that are safe, evidence-informed and used complementary to conventional medicine [11]. Complementary therapies have a wide range of health care functions and are widely used in many societies. It has been estimated that 52.5% of Iranians use complementary medicine [12].

Honey, is a promising preventive and therapeutic agent, especially against resistant organisms, thanks to its broad-spectrum antibacterial coating [13]. The safety of honey as a medical agent has been confirmed by the US Food and Drug Administration (FDA) and the Australian Therapeutic Goods Administration (TGA) [14]. Honey is known to have antibacterial effects (when honey is diluted with water, the enzyme glucose oxidase produces hydrogen peroxide, which is an effective antimicrobial substance), favorably low acidity (pH of honey is between 3.2 and 4.5, which prevents the growth of microorganisms), and osmotic effect (honey is a supersaturated sugar solution, which leaves very few water molecules for the growth of microorganisms), and also to contain antioxidants and hydrogen peroxide (the presence of various antioxidants in honey including flavonoids, phenols, polyphenols, and vitamin C, give honey an anti-inflammatory effect). These features make honey an effective natural ointment for wound dressing [9]. According to a systematic review by Saikaly et al. most of the studies conducted in the 5 year period have shown that honey is effective in healing various skin wounds [15]. In a study by Israili in the United States, the antimicrobial benefits of honey, its use in treating burns, cuts and wounds (venous, arterial, diabetic, malignancies), bedsores, postoperative infections in adults and infants, and the effectiveness of honey compounds in preventing infection at the catheter exit site in hemodialysis and peritoneal dialysis has been extensively discussed [16].

Propolis is a beehive by-product consisting of different substances, the most important of which are phenolic compounds. The main constituents of propolis are polyphenols such as phenolic acids and flavonoids, which are beneficial to human health and have medicinal and biological effects. Flavonoids are derivatives of phenylpropanoids characterized by their 15-carbon structure, which play a role in the processing of waste substances. Flavonoids also inhibit cancer cells, increase immunity (antioxidants), have antiviral, antibacterial, anti-inflammatory, and anti-allergic effects, and reduce the permeability and fragility of capillaries [17, 18]. Propolis is used to block the seams and gaps in the beehive. Researchers believe that propolis is the antibiotic of the twenty-first century [19], so propolis has many properties, including antibacterial, anti-inflammatory, antiviral, immune system stimulation, and anesthetic properties [20]. In a study by Veiga et al. in Brazil on topical treatment of chronic nail fungal infection (onychomycosis), treatment with propolis extract twice a day for 6 months had excellent clinical impacts. Accordingly, this study reported that propolis extract is a potential new therapeutic agent for the topical treatment of onychomycosis caused by trichophyton [21]. A study by Mujica et al. in Chile also reported that propolis ointment reduced the injury area by increasing the extracellular matrix and decreased the inflammation and improved the healing of diabetic foot ulcers through the enhancement of interleukin-10 [22]. Propolis have been proved to be effective and useful in not only treating, but also preventing infections [20], clinical antibacterial potential should be conducted [19].

### Objectives

This study aimed to determine the effect of propolis on the incidence of ESI and peritonitis in PD patients.

## Method

### Study design and sample

This study was a double-blind clinical trial (2019). Propolis ointment was given to dialysis patients, without specifications and labels and they did not know the type of treatment. Also, in this study, the participant, did not know they were in which group, the diagnosis of ESI and peritonitis in PD was based on ISPD findings, the clinician, laboratory specialist, and data analyzer (statistical consultant) were unaware of the arrangement of the groups. They didn’t know whether group received the intervention, the placebo group, or the control group. After completing the study and interpreting the results, the arrangement of the groups was determined to which group belonged. Participation in this study was completely voluntary. After agreeing to participate in the research, the patients were participated in a study group. They could withdraw from the research whenever they wanted, after notifying the moderator, and in case of non-participation, they would not be deprived of the usual diagnostic and therapeutic care. There was no cost for them to participate in this study. It was also explained to them that this study will continue for 6 months and once every 2 weeks, they will go to the PD unit for a free visit to check the symptoms of ESI and peritonitis, and they will be examined by a doctor, and a monthly culture will be taken from the dialysis effluent. The results of the tests will be made known to them and these results will be used completely confidentially and only for the purposes of this research, and their identities will remain confidential and will not be revealed within the framework of the law. After obtaining permission from the relevant officials, eligible subjects were selected from among PD patients who were receiving treatment in the dialysis wards. Using the block randomization method, a total of 90 patients (*n* = 90) were randomly divided into three groups: placebo (normal saline), control (mupirocin), and intervention (propolis).

Before starting the trial, patients were asked to fill out the demographic profile and informed consent forms, educated about peritoneal dialysis, how to dress the catheter exit site and apply the ointments.

### Inclusion criteria

Inclusion criteria were as follows: being aged 18–60 years, receiving continuous ambulatory peritoneal dialysis at least two times a day, elapse of at least 3 months since the start of PD, receiving no antibiotics, having no infection or peritonitis in the past month, having no skin or oral allergy to honey compounds, and completing the informed consent form (for monthly dialysate effluent smear and culture test).

### Exclusion criteria

Exclusion criteria were as follows: having other infections, having peritonitis or catheter ESI in the past month, receiving antibiotic treatment during the last 4 weeks, nasal carrying *Staphylococcus aureus*, history of mental disease, impaired perception, unwillingness to continue participation, receiving kidney transplant, shift to hemodialysis, and death.

### Intervention design

After selecting the eligible patients, first, a PD effluent smear and culture test was performed on all of them to confirm the absence of catheter infection. Also, since carriers of *Staphylococcus aureus* are more likely to develop peritonitis [6], nasal culture samples were also taken to detect these carriers, who were then treated with topical intranasal prophylaxis with mupirocin (twice daily for 5 days per month [6], before the start of the trial). Since hand washing technique, disinfection of dialysis equipment, PD technique, dressing technique, and handling of catheter can affect the likelihood of developing peritonitis, before the start of the trial, necessary education and training were provided to avoid these issues. Patients were instructed to wash the hands, prepare the PD equipment, disinfect the environment, wash the hands again, and then perform PD according to their doctor’s instructions. Dressing change was performed every other day after the morning exchange by separating the PD set, removing the catheter dressing, and checking the catheter exit site and tunnel for signs of infection (redness, pain, warmth and swelling, discharge from the catheter exit) and peritonitis. These conditions were identical in all three groups (placebo, control, and intervention), PD nurse checked the ability of the PD patients to take care of the catheter, dressing change performed after providing the training face to face, and follow-up by telephone.

In the placebo group, this process was followed by washing the catheter exit site with 0.9% normal saline, covering it with sterile gauze, and fixing the gauze with anti-allergy adhesive bandage.

In the control group, the said process was followed by washing the catheter exit site with 0.9% normal saline, using a sterile swab to rub 2% mupirocin ointment (DarouPakhsh Pharmaceutical Co. Iran) at the catheter exit site, covering it with sterile gauze, and fixing the gauze with anti-allergy adhesive bandage.

**Fig. 1:**
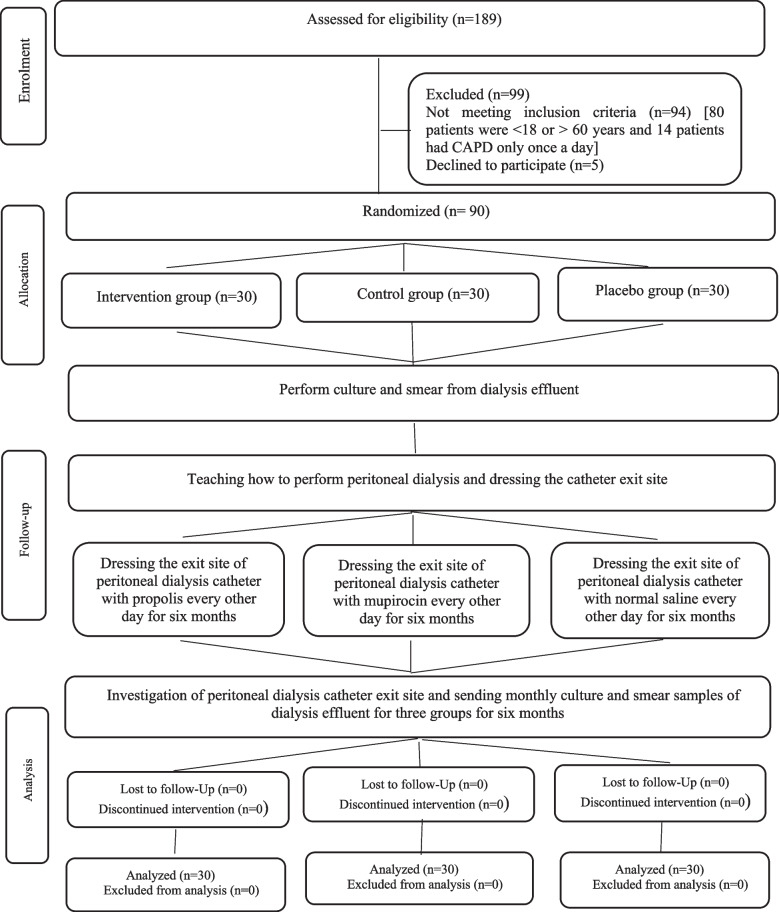
Flow diagram of the progress through the phases of a parallel-randomized trial of three groups (that is, enrollment, allocation, follow-up and analysis)

In the intervention group, the said process was followed by washing the catheter exit site with 0.9% normal saline, using a sterile swab to rub 10% propolis ointment (Dr.Jahangir Pharmaceutical & Hygienic Co. Iran) at the catheter exit site, covering it with sterile gauze, and fixing the gauze with anti-allergy adhesive bandage (Fig. 1).

All methods were carried out in accordance with relevant guidelines and regulations.

Patients were required to notify the PD nurse by telephone. Once every 2 weeks, patients returned to the PD center for the follow-up, which involved examination and checking for symptoms by a specialist and a PD nurse, and come to the PD unit immediately if any evidence of PD-related infection occurred between the clinic visits. Patients were asked to return to the PD center every month or immediately after detecting the signs of infection and peritonitis. Every month, the returning patients, after examination by a physician, PD effluent were sampled from the returning patients sent to the laboratory for culture.

### Data collection

In line with the guidelines of International Society for Peritoneal Dialysis ISPD, peritonitis was diagnosed by physician, if any two of the three following criteria were met: 1- Presence of clinical symptoms including abdominal pain and turbidity of PD effluent; 2- white blood cell count exceeding 100 with at least 50% neutrophils (polymorphonuclear) in the PD effluent; 3- positive culture of PD effluent. This diagnosis was made by a physician.

### Ethical approval

At all stages of the study, all relevant ethical and procedural requirements including those related to obtaining permit from registering the research in the Registry of Clinical Trials, [IRCT20110427006318N10] (17/01/2019) and (IR.SEMUMS.REC.1397.200). The ethical approval was obtained from Semnan University’s Ethics Committee. Informed consent was obtained from all the participants. All methods were carried out in accordance with relevant guidelines and regulations. Obtaining a letter of introduction from the university’s vice-chancellor of research, obtaining permit from the officials in charge of hospitals and PD wards, explaining the purpose of the study to patients and their guardians, reassuring patients of the confidentiality of their information, and informing patients about their right to withdraw from the study at any time were respected. The intervention did not have any side-effect and was not endangering the health of subjects, and patients were reassured of this by their physicians. Also, participation in the study was free of charge and imposed no extra cost on patients.

### Statistical analysis

The statistical analysis of demographic and background information and catheter ESI and peritonitis incidence in the three groups was carried out by the Shapiro-Wilk test, chi-square test, and one-way analysis of variance (Kruskal-Wallis). The effect of each method on the incidence of catheter ESI and peritonitis was evaluated by logistic regression. The analyses were performed using the software SPSS24.

## Results

### Sample characteristics

The mean age in the placebo group, the control group, and the intervention group were 52.4 ± 13.6 years, 55.8 ± 10.5 years, and 53.4 ± 13.3 years, respectively. The Kruskal-Wallis test showed no statistically significant difference between the groups in terms of age distribution (*P* = 0.464). The gender ratio (male/total) in both placebo and control groups was 50%, and in the intervention group was 63.3%. The three groups were not significantly different in terms of gender distribution (*P* = 0.954). The mean ± standard deviation of body mass index in the placebo group, the control group, and the intervention group was 25.8 ± 5.8 kg/m^2^, 25.4 ± 4.2 kg/m^2^, and 25.4 ± 4.2 kg/m^2^, respectively, which were not significantly different (*p* = 0.932). There was also no significant difference between the three groups in terms of education level (*p* = 0.461) or the number of PD frequency per a day (*p* = 0.194). All patients had at least one underlying disease. In placebo and control groups, 60%, and in the intervention group 76.7% of patients had hypertension. The groups were not significantly different in terms of the distribution of underlying diseases (*p* = 0.060) (Table 1).

**Table 1:** Demographic information in study groups

Demographic information		Study groups						*P*-value
		Placebo		Control		Intervention		
		N	%	N	%	N	%	
Age	< 40	4	13.3	3	10	7	23.3	0.464^a^
	40–49	7	23.3	4	13.3	2	6.7	
	50–59	6	20	8	26.7	8	26.7	
	≥ 60	13	43.3	15	50	13	43.3	
Sex	Male	19	63.3	18	60	18	60	0.954^b^
	Female	11	36.7	12	40	12	40	
Education	Primary	8	26.7	11	36.7	6	20	0.461^a^
	Secondary	15	50	15	50	21	70	
	University	7	23.3	4	13.3	3	10	
Dialysis, Frequency, Days	3	14	46.7	8	26.7	11	36.7	0.194^a^
	4	16	53.3	20	66.7	17	56.7	
	6	–	–	2	6.7	2	6.7	
Body Mass Index	< 25	16	53.3	14	46.7	15	50	0.931^C^
	25–29.9	11	36.7	12	40	8	26.7	
	≥ 30	3	10	4	13.3	7	23.3	
Co-morbidity	Hypertension	23	76.7	18	60	18	60	0.060^b^
	Diabetes	6	20	10	33.3	5	16.7	
	Others	1	3.3	2	6.7	7	23.3	
ESI	MRSE	–	–	–	–	1	3.3	
	Acinetobacter	–	–	–	–	1	3.3	
	Diphtheroids	–	–	–	–	1	3.3	
	Negative culture	–	–	2	6.7	–	–	
Peritonitis	MRSE	–	–	2	6.7	2	6.7	

a: Kruscal Wallis b: Chi square c: One-Way Anova

*MRSE* Methicillin-Resistant *Staphylococcus Epidermidis*, *ESI* Exit Site Infection

### Outcomes

#### Catheter ESI

During the first and second months, none of the patients showed catheter ESI whereas in the third month one patient (3.3%) in the placebo group showed catheter ESI with Methicillin-Resistant *Staphylococcus Epidermidis* (MRSE).

During the fourth month, one patient (3.3%) in the placebo group with Acinetobacter and one patient (3.3%) in the control group with erythema and negative culture showed catheter ESI whereas in the fifth month, only one patient (3.3%) in the placebo group presented with catheter ESI by Diphtheroids and in the sixth month one patient (3.3%) in the control group with erythema and negative culture presented with catheter ESI.

##### Catheter ESI rates for the entire 6 months

Overall, 10% (*n* = 3) of patients in the placebo group and 6.7% (*n* = 2) of patients in the control group developed catheter ESI. No patient in the intervention group developed catheter ESI. The results of logistic regression did not show any significant effect on the catheter ESI rate (*p* = 0.469) (Table 2).

**Table 2:** Incidence of peritonitis and ESI in study groups

Variables		Study groups						*P*-value
		Intervention		Control		Placebo		
		N	%	N	%	N	%	
Peritonitis	Yes	–	–	2	6.7	2	6.7	0.997
	No	30	100	28	93.3	28	93.3	
ESI	Yes	–	–	2	6.7	3	10.0	0.469
	No	30	100	28	93.3	27	90.0	

**Fig. 2:**
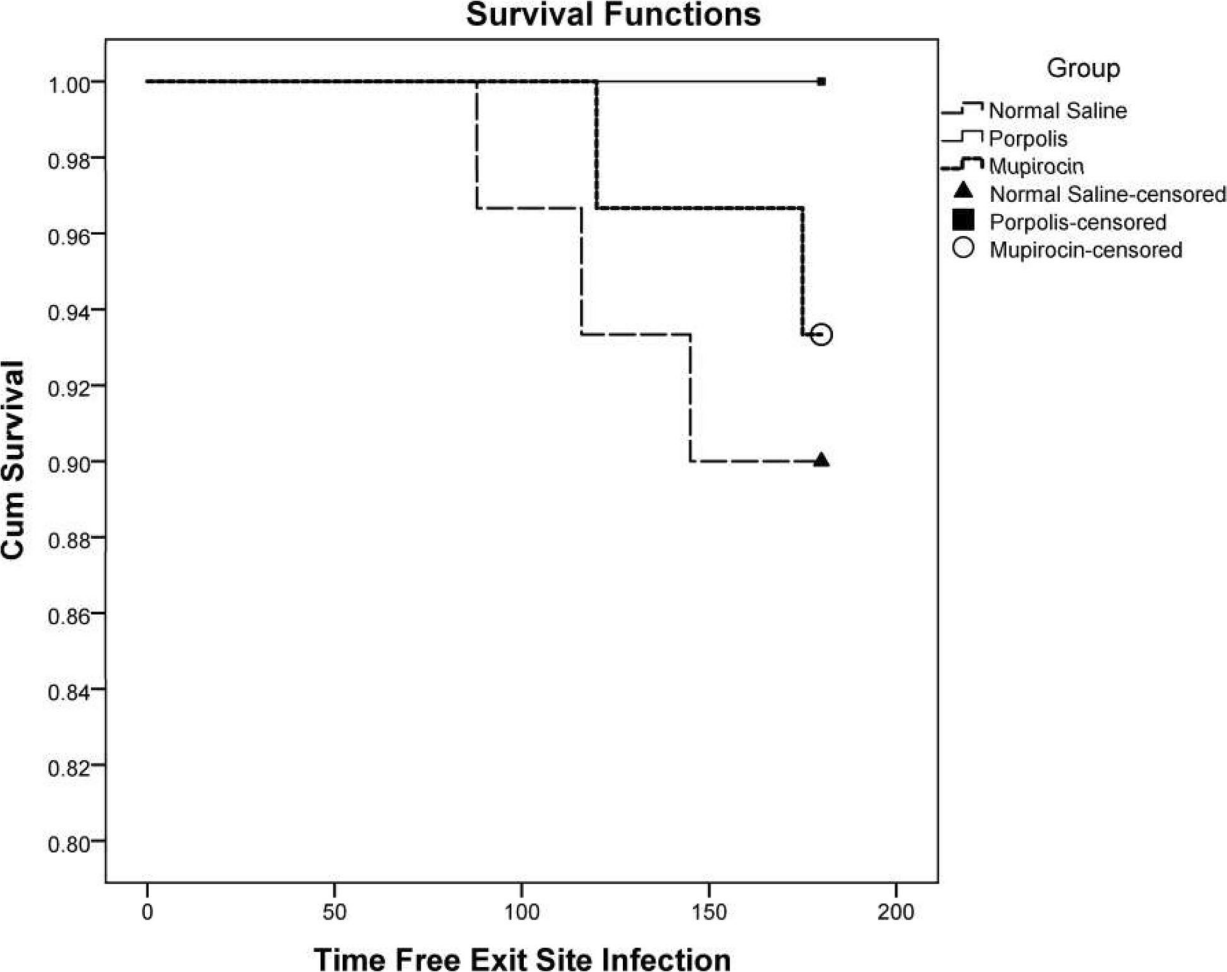
Exit Site Infection Free Survival Curve in three groups

ESI Free Survival Curve the three groups are shown in Fig. 2. There was no significant difference in the survival distribution of the three groups using the Logrank test (*p* = 0.228).

### Peritonitis

During the first 3 months, none of the patients in the three groups developed peritonitis. However, during the fourth month, one patient (3.3%) in the MRSE control group developed peritonitis (none of the variables including interventions (*p* = 1.00) showed a significant effect on peritonitis).

During the fifth month, none of the patients in the three groups developed peritonitis. Meanwhile, during the sixth month, two patients (6.7%) in the MRSE placebo group and one patient (3.3%) in the MRSE control group developed peritonitis. The results of logistic regression showed that none of the variables including interventions (*p* = 0.94) had a significant effect on the incidence of peritonitis.

#### Peritonitis rates for the entire 6 months

There was also no significant difference between the three groups in terms of the incidence of peritonitis during the 6 months of the study, Patients who developed peritonitis didn’t have ESI (Table 2). Also, no side effects were observed with the use of propolis.

**Fig. 3:**
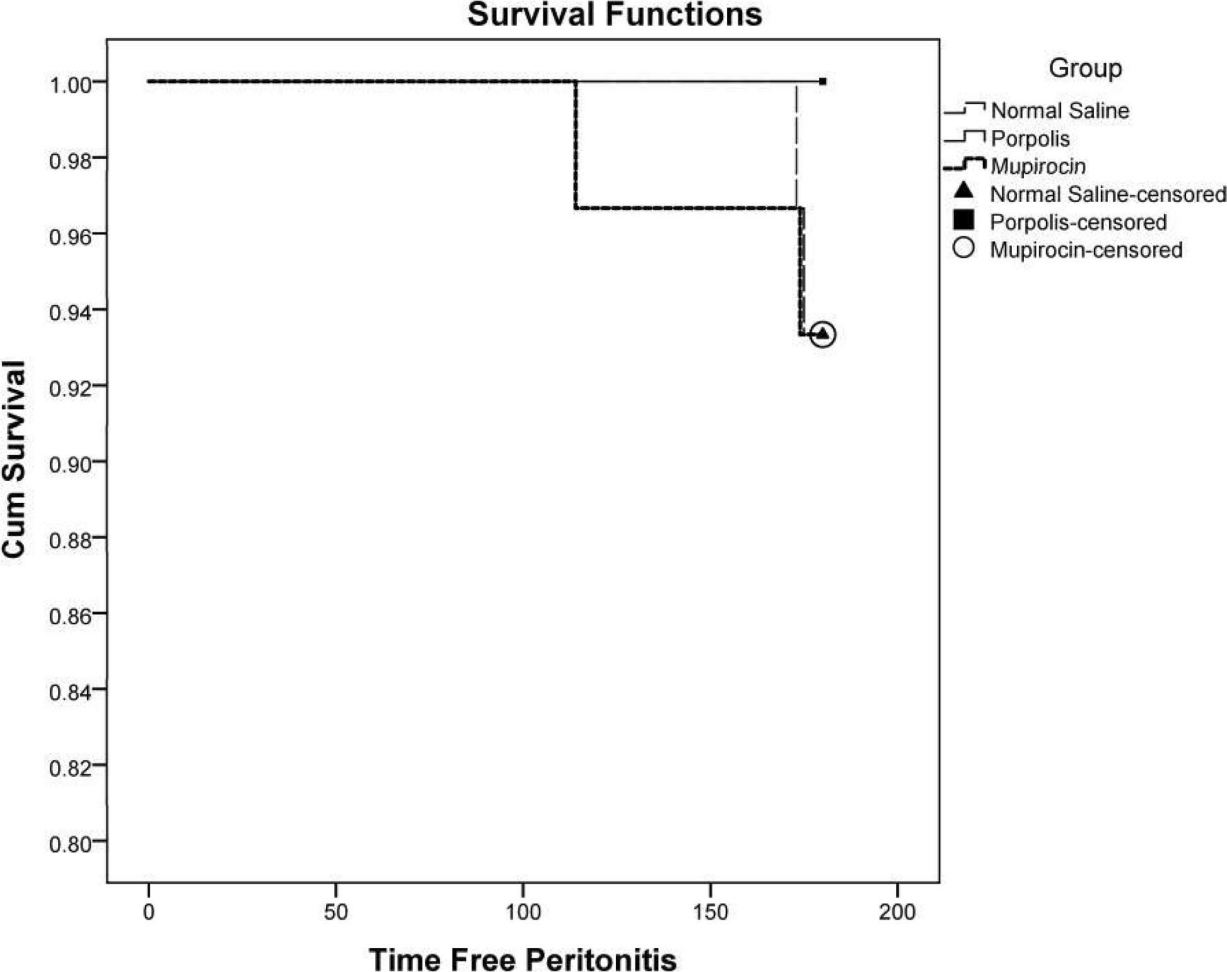
Peritonitis Free Survival Curve in three groups

Peritonitis Free Survival Curve the three groups are shown in Fig. 3. There was no significant difference in the survival distribution of the three groups using the Logrank test (*p* = 0.358).

## Discussion

This study aimed to determine the effect of propolis on catheter ESI and peritonitis in PD patients. In this study, 10% of patients (three patients) in the placebo group and 6.7% of patients (two patients) in the control group developed catheter ESI, but none of the patients in the intervention group contracted this infection. However, the results of logistic regression showed that propolis had no statistically significant impact on the incidence of catheter ESI. In a study by Nochaiwong, using mupirocin and chlorhexidine gluconate ointments rather than normal saline the risk of catheter ESI was reduced [23].

The results of logistic regression again showed that propolis had no statistically significant effect on the incidence of peritonitis. In a study by Ceri et al. ESI and peritonitis rates tended to be lower in polyhexanide group compared with the povidone-iodine group, but were not statistical significantly [24]. In a study by Wishart et al. in West Sydney, where they compared the effect of povidone-iodine and honey on PD catheter-related infections, their findings showed a higher rate of peritonitis in the povidone-iodine group than in the honey group. They reported similar findings for the overall incidence of catheter-related infections and peritonitis (*P* = 0.035) and for peritonitis alone (*P* = 0.172). These results suggest that honey is more effective than povidone-iodine and could be a safe, broad-spectrum, low-cost alternative ointment for catheter exit site [25]. Although the present study found no difference between the outcomes of using mupirocin, normal saline, or propolis, propolis can still be considered a valid preferred because of not producing antimicrobial resistance.

Similar to the present study, which found no statistically significant difference between the incidences of peritonitis and catheter ESI in placebo, control and intervention groups, a study by Zhang et al. in Australia also reported no significant difference between the groups that used mupirocin and those that used a honey-based agent in terms of incidence of gram-positive, gram-negative, and multi-organism peritonitis in PD patients [13]. Considering that unlike Zhang’s study, the present study used oil-based propolis ointment rather than honey-based antibacterial gel, the similar results of the two studies may be due to the short follow-up period of this study (6 months) compared to Zhang’s (24 months) [13].

The most important limitation of this study was difficulty in recruiting patients because of their doubts about the intervention, but this issue was largely resolved by educating the patients both directly and through their physicians. Another limitation was small sample size. Nevertheless, it is recommended to conduct a similar study with a larger sample and a longer follow up period.

Considering that peritonitis is one of the major complications of peritoneal dialysis, ISPD has recommended the daily use of antibacterial ointments, including mupirocin, to prevent catheter ESI and peritonitis. However, there are reports of resistance to mupirocin after regular use [26, 27]. While mupirocin is the most recommended topical antibiotic for use at the catheter exit site, it reduces the likelihood of developing *Staphylococcus aureus* peritonitis by only 40% [27]. Also, the extensive use of mupirocin will inevitably lead to the emergence of resistant organisms [13]. In view of these issues and also the potential side effects of using chemical antibacterial agents to prevent catheter infection in PD patients, it may be worthwhile to introduce new treatments with lower side effected for these patients. Considering the findings of this study and the novelty of the subject, and the fact that mupirocin causes antimicrobial resistance and is more expensive than propolis, there is some room to hope that using propolis as alternative medicine may reduce treatment costs and help avoid causing antimicrobial resistance.

In general, the findings did not show a significant difference among the three groups in terms of incidence of catheter ESI and peritonitis. Considering that mupirocin is of chemical origin and may lead to drug resistance whereas propolis is effective against infections, instead of mupirocin or other agents to prevents ESI and peritonitis, propolis is more convenient, patient-friendly, cost-effective and easily available for PD patients. More studies with larger number of patients enrolled in future studies, being defined by a power calculation execution, as well as longer interventional time is needed.

## Data Availability

The datasets generated and analyzed during the current study are not publicly available due to an agreement with the participants on the confidentiality of the data but are available from the corresponding author on reasonable request.
